# Common Variable Immunodeficiency with Genetic Defects Identified by Whole Exome Sequencing

**DOI:** 10.1155/2018/3724630

**Published:** 2018-09-30

**Authors:** Ran Li, Yali Zheng, Yuqian Li, Rongbao Zhang, Fang Wang, Donghong Yang, Yanliang Ma, Xinlin Mu, Zhaolong Cao, Zhancheng Gao

**Affiliations:** Department of Respiratory and Critical Care Medicine, Peking University People's Hospital, Beijing 100044, China

## Abstract

Common variable immunodeficiency (CVID) belongs to the primary immunodeficiency disorders (PIDs), presenting a profound heterogeneity in phenotype and genotype, with monogenic or complex causes. Recurrent respiratory infections are the most common clinical manifestations. CVID patients can also develop various autoimmune and lymphoproliferative complications. Genetic testing such as whole exome sequencing (WES) can be utilized to investigate likely genetic defects, helping for better clinical management. We described the clinical phenotypes of three sporadic cases of CVID, who developed recurrent respiratory infections with different autoimmune and lymphoproliferative complications. WES was applied to screen disease-causing or disease-associated mutations. Two patients were identified to have monogenic disorders, with compound heterozygous mutations in* LRBA* for one patient and a frameshift insertion in* NFKB1* for another. The third patient was identified to be a complex form of CVID. Two novel mutations were identified, respectively, in* LRBA* and* NFKB1*. A combination of clinical and genetic diagnosis can be more extensively utilized in the clinical practice due to the complexity and heterogeneity of CVID.

## 1. Introduction

Common variable immunodeficiency (CVID) is the most common primary immunodeficiency disorder (PID) with an estimated prevalence of 1:50,000 to 1:25,000 [1]. CVID is characterized by remarkable hypogammaglobulinemia resulting from B-lymphocyte dysfunction, which also involves T-lymphocyte abnormalities [2]. CVID is usually complicated by recurrent infections, autoimmune diseases, malignancies, and lymphoproliferative diseases. Pulmonary infections are most commonly developed in CVID patients, which may further lead to bronchiectasis. Noninfectious pulmonary complications exhibit as granulomatous-lymphocytic interstitial lung disease (GLILD). Multiple systems can be involved as a result of immune dysregulation, such as cytopenia, splenomegaly, enteropathy, and various autoimmune diseases.

CVID shows a considerable heterogeneity in phenotype and genotype. Most cases of CVID occur sporadically, with approximately 5%~25% of patients having a familial tendency however [3]. Most cases have an unknown genetic cause, with monogenic diseases accounting for only 2%~10% with both autosomal recessive and dominant inherited mutations [3]. Monogenic defects that have been implicated include recessively inherited mutations in* ICOS*,* CD19*,* CD20*,* CD21*,* CD27*,* CD81*,* IL21*,* IL21R*,* LRBA*,* PRKCD*, and* RAC2*, and dominantly inherited mutations in* TNFSF12*,* CTLA4*,* PLCG2*,* NFKB1*,* NFKB2*,* PIK3CD*,* PIK3R1*,* VAV1*,* BLK*,* IKZF1,* and* IRF2BP2*, as well as monoallelic or biallelic mutations in* TNFRSF13B* and* TNFRSF13C* [4]. A subgroup of patients demonstrates a complex disorder, rather than a Mendelian inheritance, with possible polygenic, epigenetic, and/or environmental factors participating in CVID pathogenesis.

Genetic testing is applied to unravel the heterogeneity of CVID, including genetic linkage studies in families, genomewide association study (GWAS), whole genome sequencing (WGS), whole exome sequencing (WES), RNA sequencing, and epigenetic studies [5, 6]. CVID patients may benefit from genetic testing to identify harmful variations and allow genetic counseling. WES is a potential effective tool to help discover genetic defects for monogenic cases, which targets protein-coding sequences accounting for 1% of the whole genome, but reported to harbor 85% of disease-causing variants [7]. Here we describe the phenotypes of three sporadic CVID cases admitted to the Department of Respiratory and Critical Care Medicine, Peking University People's Hospital, and further investigate the genetic disorders using the approach of WES.

## 2. Materials and Methods

### 2.1. Patient Phenotypes

**Figure 1:**
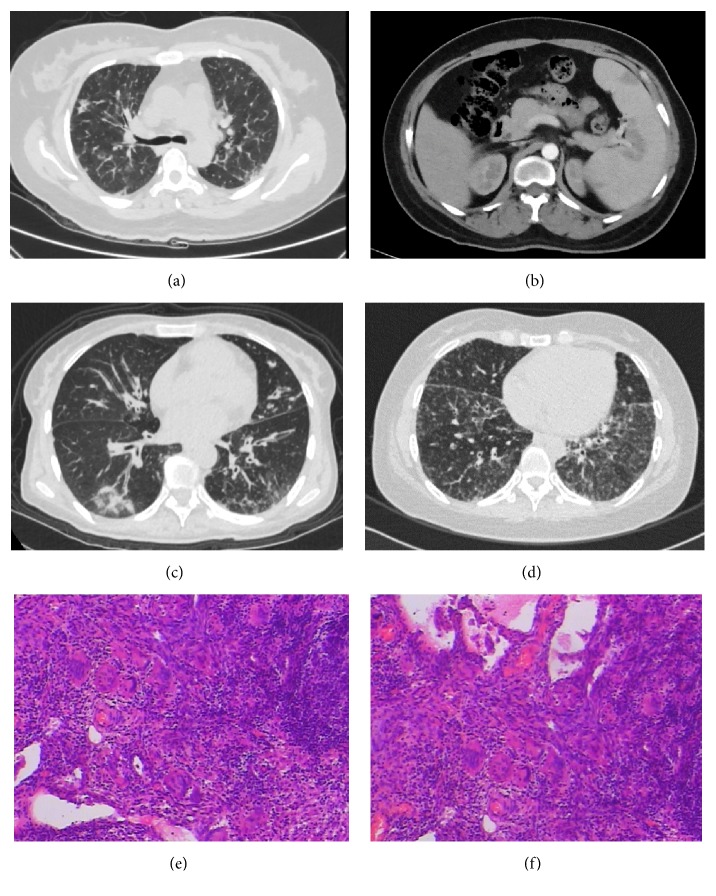
CT appearance of the three cases and pathological features of case 3. Panel (a) showed diffused reticulation as the manifestation of suspected GLILD in case 1. Panel (b) showed focal hypodense splenic lesions with splenomegaly in case 1. Panel (c) showed bronchiectasis with infiltrates and mucus plugs in case 2. Panel (d) showed diffused micronodules and bronchiectasis as the manifestation of GLILD in case 3. Panels (e) and (f) revealed diffused infiltration of lymphocytes and lymphoid follicles formation in the lung tissues, scattered with epithelioid granulomas and multinuclear giant cells, consistent with the pathological manifestation of GLILD.

Case 1, female, 46 years old, presented with a history of recurrent lower respiratory tract infections at the age of 38. She developed suppurative meningitis at the age of 43, with a sequela of blindness. Other clinical conditions included chronic sinusitis, pancytopenia, splenomegaly, and sensorineural hearing loss. Immunological findings showed decreased levels of IgG, IgA, and IgM in serum and a low proportion of B cells (Table 1). GLILD was suspected according to the chest CT without a pathologic confirmation (Figure 1(a)). Pulmonary function tests (PFTs) demonstrated a mild restrictive ventilatory defect and a diffusion impairment. Abdominal contrast-enhanced CT revealed multiple hypodense lesions in the spleen, which mimicked splenic infarction (Figure 1(b)).

Case 2, female, 54 years old, presented with a history of recurrent lower respiratory tract infections at the age of 39. She suffered from tuberculous pleuritis at the age of 46. Other clinical conditions included chronic sinusitis, intermittent gastrointestinal infections, neutropenia, sensorineural hearing loss, and splenomegaly. The patient had extremely low levels of IgG, IgA, and IgM, a low proportion of B cells, and an inverted CD4^+^/CD8^+^ ratio (Table 1). Chest CT showed bilateral bronchiectasis with multiple infiltrates (Figure 1(c)). PFTs demonstrated a severe obstructive ventilatory defect and a diffusion impairment.

Case 3, female, 34 years old, presented as recurrent lower respiratory tract infections with an onset age of seven. Other clinical conditions included autoimmune hemolytic anemia, splenomegaly, hypothyroidism, and nephrotic syndrome. Decreased levels of IgG, IgA, and IgM and an inverted CD4^+^/CD8^+^ ratio were also detected (Table 1). Chest CT demonstrated diffused nodules, bronchiectasis, and mediastinal lymphadenopathy (Figure 1(d)), with a severe restrictive ventilatory defect and a diffusion impairment confirmed by PFTs. Wedge resection of the right middle lobe and right lower lobe was performed; the diagnosis of GLILD was confirmed by pathology subsequently (Figures 1(e) and 1(f)).

The three cases met the criteria for CVID established by European Society for Immunodeficiencies/Pan-American Group for Immunodeficiency [8]. All the cases received antibiotics and immunoglobulin replacement therapy and survived to date. Case 3 received extra corticosteroids treatment for GLILD with clinical improvement. Normal IgG, IgA, and IgM levels were detected in the offspring of the probands, including the daughter and the son of case 1, the son of case 2, and the son of case 3.

### 2.2. Whole Exome Sequencing

Genomic DNA of the patients and offspring was isolated from peripheral blood mononuclear cells. The protocol was approved by the Ethics Committee of Peking University People's Hospital, and informed consent was obtained from the subjects. The exome sequences were efficiently enriched from 1.0 *μ*g genomic DNA using the Agilent SureSelect Human All Exon V6 kit (Agilent Technologies). Qualified DNA was randomly fragmented to an average size of 180~280 bp; then DNA fragment was end-repaired and phosphorylated, followed by A-tailing and ligation at the 3' ends with paired-end adaptors (Illumina). At last, DNA library was sequenced on a Hiseq 4000 (Illumina) for paired-end 150 bp reads. Valid sequencing data were mapped to the reference genome (UCSC hg19) by Burrows-Wheeler Aligner software (BWA) [9]. SAMtools [10] and Picard Tools (http://broadinstitute.github.io/picard/) were utilized to sort the results and mark duplicate reads, respectively. Single nucleotide variants (SNVs) and insertions and deletions (Indels) detected were annotated with the ANNOVAR software (http://annovar.openbioinformatics.org/en/latest/) [11].

### 2.3. Candidate Gene Screening

Patients' exomes were filtered for mutations associated with immunodeficiency [12]. Synonymous SNVs were discarded. SNVs obtained with the minor allele frequency (MAF)<0.01 in the general population according to the Exome Aggregation Consortium database (ExAC, Broad Institute) were supposed to be novel or rare, which were potentially significant. Deleterious variations were predicted utilizing SIFT, PolyPhen-2, MutationTaster, and CADD [13–16]. Afterwards, candidate variants were screened based on the phenotypes and the inheritance patterns of the patients. Deleterious indels associated with the phenotypes were also screened. As the three cases were all sporadic without a familial inheritance tendency, we firstly hypothesized that the patients had monogenic disorders with an autosomal recessive pattern caused by a homozygous or compound heterozygous inheritance or with a dominant pattern with an incomplete penetrance. If no causative mutations were found, we considered the case as a complex form of CVID rather than a Mendelian disease. Top likely disease-associated mutations were confirmed by Sanger sequencing.

## 3. Results

**Figure 2:**
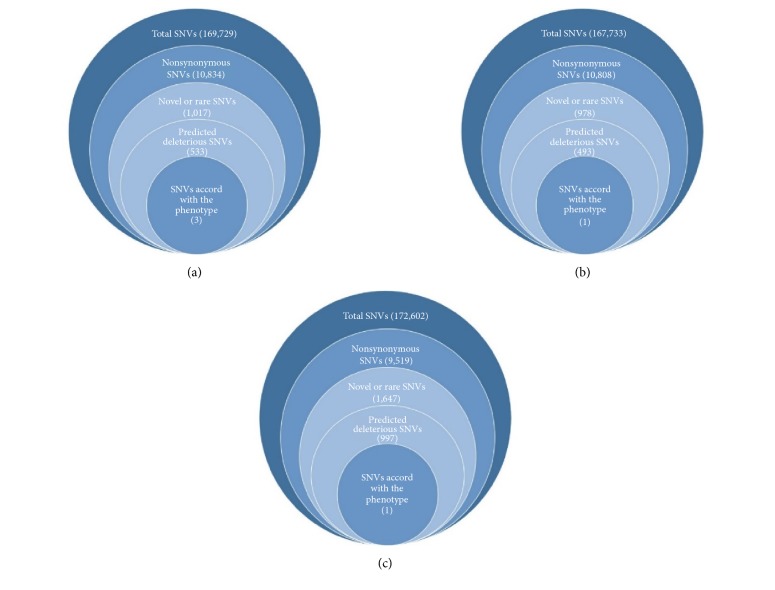
Filtering strategies for candidate SNVs in the three cases, with panel (a) for case 1, panel (b) for case 2, and panel (c) for case 3. The number of genes filtered was within the parentheses.

The filtering results for candidate SNVs were shown in Figure 2. Three SNVs in case 1, one SNV in case 2, one SNV, and one insertion in case 3 were most likely to be disease-associated (Table 2). All the candidate mutations have been confirmed by Sanger sequencing (supplementary figure (available here)).

In case 1, heterozygous variants in* LRBA *(c.8436G>C and c.4089A>T) and in* TNFRSF13B *(c.226G>A) were identified to be disease-associated. LRBA deficiency was reported to be recessively inherited accounting for 26.74% of monogenic causes of CVID [4, 12, 17]. Further sequencing of her offspring showed biallelic variants in* LRBA* of the proband were separately inherited by her unaffected daughter (c.8436G>C) and her unaffected son (c.4089A>T), demonstrating the* LRBA* mutations were probably compound heterozygous which led to CVID. The variant of c.4089A>T is novel and has not been reported before. Biallelic and monoallelic* TNFRSF13B *variants are both reported to be disease-associated [4, 18]. However, the* TNFRSF13B *variant in this patient alone is insufficient to cause a CVID phenotype, which may play a role in CVID development but was not crucial. We also found that the* TNFRSF13B* variant was inherited by her unaffected daughter. Therefore, we identified compound heterozygous mutations in* LABA *most likely to be disease-causing in this patient.

In case 2, a heterozygous* LRBA* variant (c.3764G>C) was filtered out. However, a monoallelic* LRBA* mutation cannot explain the phenotype of the patient. As a result, we assumed case 2 was a complex form of CVID rather than a monogenic disease, although the monoallelic variant in* LRBA* may play a minor role. Further investigations found that the* LRBA* variant was not transmitted to her unaffected son.

In case 3, a heterozygous* LRBA *variant (c.5084T>C) was identified, which was inherited by her unaffected son. Likewise, this monoallelic* LRBA* mutation was insufficient to explain a disease-causing effect. We also found a monoallelic insertion in* NFKB1 *(c.666dupG), resulting in a frameshift mutation. Considering mutations in* NFKB1 *were reported to be inherited with an autosomal dominant trait [4], the insertion in* NFKB1 *was considered to be causative in case 3. This* NFKB1* insertion is also novel and has not been reported before. Since mutations in* NFKB1 *were inherited with an incomplete penetrance [4], her son also inherited the mutation without a clinical phenotype of CVID.

Overall, two patients with monogenic causes and one patient with a complex cause of CVID were identified using WES. Two novel mutations were found, respectively, in* LRBA* (c.4089A>T) and in* NFKB1 *(c.666dupG).

## 4. Discussion

The three cases of CVID we discussed had different clinical phenotypes with different genetic defects. Respiratory tract infections and various noninfectious complications were noted, with GLILD suspected or confirmed in two cases. The radiological abnormalities in CVID patients with GLILD mainly manifest as diffuse reticulation or nodules, while isolated bronchiectasis can also be seen [19]. Lymphadenopathy is sometimes accompanied by interstitial changes, and lymphoma should be excluded, with a higher risk in CVID patients [20]. The type of impaired lung function was consistent with the radiological findings in our cases, with interstitial lung disease (ILD) outweighing the impact of bronchiectasis in case 3. GLILD exhibits both granulomatous and lymphoproliferative patterns in histologic examinations, consisting of lymphocytic interstitial pneumonia, follicular bronchiolitis, and lymphoid hyperplasia, with a potential role of human herpesvirus 8 in the pathogenesis [21]. At present, GLILD is defined as a distinct clinico-radio-pathological ILD in CVID patients, with corticosteroids as the first-line treatment [22]. Splenomegaly in our patients was also a manifestation of the lymphoproliferative and granulomatous disease. Hypodense splenic lesions were considered nonspecific with a nonneoplastic etiology, since spontaneous resolution was reported for similar lesions [23]. Recognition of the noninfectious complications, which is not completely understood currently, is important for including an accurate evaluation for the disease, as noninfectious complications reduce the overall survival in CVID patients [24, 25].

As an increased number of genetic defects was found, genetic diagnosis is more and more important in PID classification, because of the broad overlapping in clinical and immunological features. With the help of genetic testing, the diagnosis of CVID could be more precise, such as LRBA deficiency or NF*κ*B1 deficiency. WES was reported to identify 30% of disease-causing mutations in CVID patients with severe phenotypes [26].

Deleterious* LRBA* mutations were found in all three patients. Lipopolysaccharide-responsive beige-like anchor protein (LRBA) is a cytosolic protein expressed by immune effector cells. LRBA participates in the CTLA-4 pathways, which negatively regulates immune responses [27]. LRBA deficiency results in a loss of CTLA-4 protein. Hence, LRBA deficiency usually results in immune dysregulation and autoimmunity in CVID patients.* LRBA* mutations were also associated with inflammatory bowel disease-like disorder, and immune dysregulation, polyendocrinopathy, enteropathy, and X-linked-like disease [28, 29]. Homozygous mutations in* LRBA* were reported to result in loss of function in multiple consanguineous families [17], while compound heterozygous mutations can also cause a CVID phenotype as we identified in case 1 [26]. Monoallelic* LRBA* mutations are insufficient to be disease-causing and reported to be disease-associated, causing recurrent pulmonary infections, organomegaly, and autoimmune cytopenia [26]. An explanation is that a monoallelic mutation may also influence the protein stability of LRBA [30].

*TNFRSF13B* belongs to the tumor necrosis factor receptor (TNFR) superfamily, which encodes transmembrane activator and calcium-modulator and cyclophilin ligand interactor (TACI) and plays a vital role in the maturation and survival of peripheral B cells [31]. Heterozygous variations in* TNFRSF13B *are disease-modifying mutations rather than disease-causing mutations, which may increase the risk for developing CVID and are also found in healthy individuals [4, 18, 32]. The* TNFRSF13B* mutations can participate in the pathogenesis of CVID through the epistatic interactions with mutations of other genes [33]. In addition, the* TNFRSF13B *mutations were reported to have an incomplete penetrance, which may explain the different phenotypes between family members harboring the same variants [4, 31, 34, 35].

NF-*κ*B signaling pathways participate in the process of B cell differentiation and function, playing a pivotal role in the pathogenesis of multiple diseases of immune dysfunction [36, 37].* NFKB1 *encodes the mature p52 subunit and its precursor p105 subunit, which belongs to the NF-*κ*B transcription factor family, reported to be associated with CVID in multiple consanguine families or sporadic cases [26, 37].* NFKB1 *mutations have an autosomal dominant inheritance with an incomplete penetrance, since the variants were also found in unaffected family members [37].

Our study has some limitations. Firstly, the functional effects of the found mutations are not verified at the expression level but have provided additional information for clinical management. Secondly, since the parents of the probands were unavailable for genetic testing, the interpretation for the mode of inheritance and gene functions is limited.

In conclusion, we described the clinical phenotypes of three sporadic cases of CVID and tried to identify genetic defects in these patients using WES. Since CVID forms a heterogeneous group of phenotypes and genotypes, genetic testing promotes the diagnosis of CVID to the genetic level, as well as profoundly improves our understanding for CVID. A combination of clinical and genetic diagnosis can be more extensively utilized in the clinical practice of CVID. However, because of the complexity of the disease, genetic investigation is still a great challenge.

## Figures and Tables

**Table 1:** Immunological phenotypes of the three cases.

**Case**	**IgG**	**IgA**	**IgM**	**B cells**	**C** **D**4^+^/**CD**8^+^
	**(7.2** **~16.8 g/L)**	**(0.82** **~4.53 g/L)**	**(0.46** **~3.04 g/L)**	**(9**%**~29**%**)**	**(0.71** **~2.78)**
**1**	3.9	<0.07	0.068	1.7%	1.16
**2**	<0.3	<0.07	<0.042	2.81%	0.35
**3**	0.488	0.0667	0.258	18.71%	0.48

**Table 2:** Mutations likely associated with CVID in the three cases.

**Case**	**Gene**	**dbSNP ID**	**mRNA Refseq**	**Coding ** **change**	**Protein change**	**Functional ** **effect**	**ExAC**	**SIFT/PolyPhen/ ** **MutationTaster/CAD** **D** ^**∗**^
1	*LRBA*	rs200809013	NM_006726	c.8436G>C	p.K2812N	missense	0.001	T/P/D/25.2
		-	NM_006726	c.4089A>T	p.Q1363H	missense	-	D/P/D/27.0
	*TNFRSF13B*	rs146436713	NM_012452	c.226G>A	p.G76S	missense	0.0002	D/D/D/28.6
2	*LRBA*	rs191899647	NM_006726	c.3764G>C	p.R1255T	missense	0.00005789	D/B/D/15.06
3	*NFKB1*	-	NM_003998	c.666dupG	p.P222fs	frameshift	-	-
						insertion		
	*LRBA*	rs200935054	NM_006726	c.5084T>C	p.V1695A	missense	0.00007419	T/B/D/16.84

^*∗*^Using SIFT, PolyPhen, MutationTaster, and CADD to predict deleterious SNVs. SIFT (T, tolerated; D, deleterious); PolyPhen (D, probably damaging; P, possibly damaging; B, benign); MutationTaster (D, disease-causing); CADD (score>15 implied deleterious variations).

## Data Availability

The data used to support the findings of this study are available from the corresponding author upon request.

## Supplementary Materials

Supplementary Materials Supplementary figure. Results of Sanger sequencing for mutations likely associated with the disease. A.* LRBA* variant (c.8436G>C) in case 1; B.* LRBA* variant (c.4089A>T) in case 1; C.* TNFRSF13B* variant (c.226G>A) in case 1; D.* LRBA* variant (c.3764G>C) in case 2; E.* LRBA* variant (c.5084T>C) in case 3; F.* NFKB1* variant in case 3 (c.666dupG).
