# Supplementary material for: Common Variable Immunodeficiency with Genetic Defects Identified by Whole Exome Sequencing
Source: Biomed Res Int. 2018 Sep 30;2018:3724630. doi: 10.1155/2018/3724630 (PMC6186323; doi:10.1155/2018/3724630)
Supplement: Supplementary Materials — Supplementary figure. Results of Sanger sequencing for mutations likely associated with the disease. A. LRBA variant (c.8436G>C) in case 1; B. LRBA variant (c.4089A>T) in case 1; C. TNFRSF13B variant (c.226G>A) in case 1; D. LRBA variant (c.3764G>C) in case 2; E. LRBA variant (c.5084T>C) in case 3; F. NFKB1 variant in case 3 (c.666dupG). [file 3724630.f1.docx]

**Supplementary Materials**

| 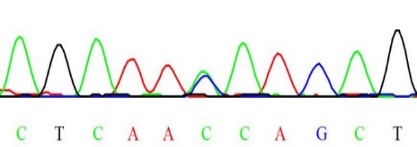 A | 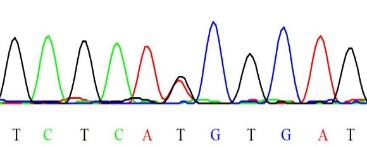  B | 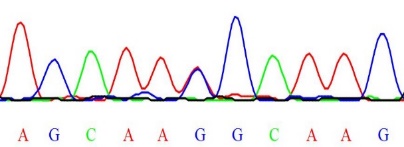  C |
| --- | --- | --- |
| 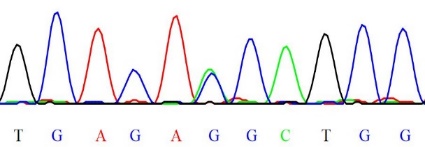  D | 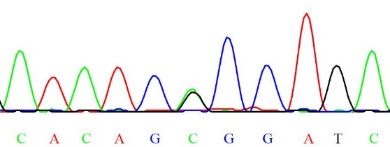  E | 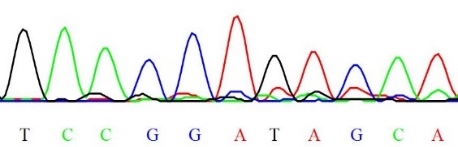F |

Supplementary figure. Results of Sanger sequencing for mutations likely associated with the disease. A. *LRBA* variant (c.8436G＞C) in case 1; B. *LRBA* variant (c.4089A＞T) in case 1; C. *TNFRSF13B* variant (c.226G＞A) in case 1; D. *LRBA* variant (c.3764G＞C) in case 2; E. *LRBA* variant (c.5084T＞C) in case 3; F. *NFKB1* variant in case 3 (c.666dupG).
